# Selection of appropriate reference genes for the detection of rhythmic gene expression via quantitative real-time PCR in Tibetan hulless barley

**DOI:** 10.1371/journal.pone.0190559

**Published:** 2018-01-08

**Authors:** Jing Cai, Pengfei Li, Xiao Luo, Tianliang Chang, Jiaxing Li, Yuwei Zhao, Yao Xu

**Affiliations:** 1 Provincial Key Laboratory of Biotechnology of Shaanxi Province, Xi’an, China; 2 Life Sciences School of Northwest University, Xi’an, China; 3 Key Laboratory of Resource Biology and Biotechnology in western China (Ministry of Education), Xi’an, China; University of Western Sydney, AUSTRALIA

## Abstract

Hulless barley (*Hordeum vulgare* L. var. *nudum*. hook. f.) has been cultivated as a major crop in the Qinghai-Tibet plateau of China for thousands of years. Compared to other cereal crops, the Tibetan hulless barley has developed stronger endogenous resistances to survive in the severe environment of its habitat. To understand the unique resistant mechanisms of this plant, detailed genetic studies need to be performed. The quantitative real-time reverse transcription-polymerase chain reaction (qRT-PCR) is the most commonly used method in detecting gene expression. However, the selection of stable reference genes under limited experimental conditions was considered to be an essential step for obtaining accurate results in qRT-PCR. In this study, 10 candidate reference genes—*ACT* (Actin), *E2* (Ubiquitin conjugating enzyme 2), *TUBα* (Alpha-tubulin), *TUBβ6* (Beta-tubulin 6), *GAPDH* (Glyceraldehyde 3-phosphate dehydrogenase), *EF-1α* (Elongation factor 1-alpha), *SAMDC* (S-adenosylmethionine decarboxylase), *PKABA1* (Gene for protein kinase HvPKABA1), *PGK* (Phosphoglycerate kinase), and *HSP90* (Heat shock protein 90)—were selected from the NCBI gene database of barley. Following qRT-PCR amplifications of all candidate reference genes in Tibetan hulless barley seedlings under various stressed conditions, the stabilities of these candidates were analyzed by three individual software packages including geNorm, NormFinder, and BestKeeper. The results demonstrated that *TUBβ6*, *E2*, *TUBα*, and *HSP90* were generally the most suitable sets under all tested conditions; similarly, *TUBα* and *HSP90* showed peak stability under salt stress, *TUBα* and *EF-1α* were the most suitable reference genes under cold stress, and *ACT* and *E2* were the most stable under drought stress. Finally, a known circadian gene *CCA1* was used to verify the service ability of chosen reference genes. The results confirmed that all recommended reference genes by the three software were suitable for gene expression analysis under tested stress conditions by the qRT-PCR method.

## Introduction

The continual growth of populations and excessive use of chemical fertilizers in agricultural productivities have remarkably and negatively influenced the natural environment: arable lands have decreased, air and water have been polluted, severe desertification has increased, global warming is changes the climate, and frequent natural disasters threaten plant growth [1, 2]. According to the characteristics of these stress factors, they can be classified into two categories of environmental stresses to organisms: biotic and abiotic stresses. Among all abiotic stress factors—such as drought, high temperatures, cold, UV radiation, and mechanical injury—salt, cold, and drought stresses are the most regular abiotic stresses, which may be encountered by crops throughout their life spans and which also negatively affect growth, yield, and quality of cereal crops [3–5]. The focus of research has always been on the abiotic stresses of plants [6]. Owing to the rapid development of the new generation of sequencing technology, a variety of biological genome sequencing studies have been completed. The genome sequences of many important crops have also been recently reported, such as rice and barley [7, 8]. These studies have accelerated the development of genomics and comparative genomics research in crops enormously, and provided a shortcut for revealing the constitution and regulations of plant stress-resistance mechanisms in different species via comparative genomics method.

Tibetan hulless barley (*Hordeum vulgare* L. var. *nudum*. hook. f.) is a type of traditional crop in the Qinghai-Tibetan plateau, which has been cultivated as an irreplaceable cereal by the local inhabitants for hundreds of years. As a true functional food, the hulless barley flour is rich in dietary fiber, which significantly reduces the risk of type II diabetes, cardiovascular disease, and colorectal cancers [7, 9–12]. As a result of its very high altitude, the environment of the Qinghai-Tibetan plateau has been believed to be one of the harshest conditions for agriculture on our planet. Compared with the normal cultivated barley and all other plants within the genus *Hordeum*, hulless barley has evolved stronger endogenous resistance systems to resist the intensive UV radiation, lower oxygen pressure, and other abiotic stresses, such as salt, cold, and drought stresses, to better adapt to the severe environmental conditions of this area [13]. Still, the mechanism of how hulless barley stress defense systems are organized and the expression profiles of most key stress-resistant genes remained unknown for this plant. Further, as a diploid, hulless barley can also serve as a new model species for the genetic research within the genus *Hordeum* [7]. Recently, with the rapid development of molecular biology technology, crops have been multiple genome-wide sequenced, providing a solid foundation to investigate the molecular mechanism of plant resistance features that has been thought to be controlled by multiple genes [7, 13, 14].

At present, the detection of gene expression profiles in plants is usually conducted via Northern blotting, competitive RT-PCR, microarray, or qRT-PCR techniques. All these commonly used methods have shown shortcomings, such as Northern blot being an experience-requiring and time-consuming technique [15], while the selection of appropriate internal standard RNAs significantly affects the accuracy of competitive RT-PCR experiments [16]. To ensure the veracity and repeatability of experiments, high-throughput methods, such as microarrays or RNA-seq, are always expensive and not only require high quality, but also a significant amount of RNA samples [17]. Compared to these methods, qRT-PCR has many advantages, such as high sensitivity, high specificity, high accuracy, ease of operation, and lower consumption of supplies. On account of these features of qRT-PCR, it is more convenient for it to be used as a rapid detection protocol for the mRNA titers or gene expression among limited numbers of sample groups [18, 19], either in different species [20, 21], in different developmental stages [22, 23], or in various responding processes to abiotic and biotic stresses [14, 24]. A primary requirement for the detection of the gene expression quantity is that the total amount of RNAs of individual samples should be equal. Qualified reference genes have often been employed as internal standards for volume calibration of RNA samples, which always played a pivotal role in obtaining reliable quantitative analysis data from genes expression [25, 26]. The use of optimized reference genes remarkably reduces experimental errors that have been generated during the sample preparing procedure. Housekeeping genes of plants, which have usually been constantly expressed in most of the developmental stages during their life spans, are preferred candidates for reference genes in qRT-PCR experiments [23, 27]. However, the expression profiles of most generally-considered housekeeping genes would be influenced by changes in environmental conditions, especially by being induced or suppressed by environmental stresses to various degree [23, 28]. Recent studies have proven that no tested genes could be used as universal reference gene under all circumstances [18, 29]. This means the expression profiles of optimized reference genes can only be chosen to meet one of the following requirements, such as to be constant in a manner of environmental condition-specific, tissue-specific, or developmentally specific, as well as in cultivar-dependent manners [30–32]. Therefore, to accurately detect expression patterns of functional genes in plants under various stress conditions, it is necessary to select optimized reference genes whose expression remains constant under certain stresses [25, 26].

In this present study, 10 candidate reference genes from hulless barley (as shown in Table 1)—*ACT* (*Actin*) [33], *E2* (*Ubiquitin conjugating enzyme 2*) [34], *TUBα* (*Alpha-tubulin*), *TUBβ6* (*Beta-tubulin 6*) [35], *GAPDH* (*Glyceraldehyde 3-phosphate dehydrogenase*) [36], *EF-1α* (*Elongation factor 1-alpha*) [37], *SAMDC* (*S-adenosylmethionine decarboxylase*) [38], *PKABA1* (*Gene for protein kinase HvPKABA1*) [39], *PGK* (*Phosphoglycerate kinase*) [40], and *HSP90* (*Heat shock protein 90*) [41]—were chosen as candidates to evaluate expression stability via qRT-PCR. Three popular software packages, including geNorm, NormFinder, and BestKeeper, were employed to conduct the following data analysis [35, 42–44]. The performance of most suitable reference genes were then tested with a time course expression pattern of the well-studied clock gene, *CIRCADIAN CLOCK-ASSOCIATED 1* (*CCA1*) (JN603242.1) [45–48]. This study has achieved optimal reference genes for hulless barley under individual cold, salt, and drought stresses. The results provided a solid experimental basis for further research either on subsequent functional gene expression pattern analysis of hulless barley or for the exploration of the mechanism with which this crop has adapted to the harsh environments of its habitats.

**Table 1:** Description of primer sequences and amplicon characteristics in ten candidate reference genes.

Gene	Gene description	Primer sequence S/A(5'-3')	Product size(bp)	TM(°C)	Effciency(%)	R^2^	Mean Ct	SD	CV(%)
*EF-1α*	Elongation factor 1-alpha	CCAACTTCACTGCCCAGGTCA	293	56	97	0.999	22.34	1.78	7.96
		CACAGCAACCGTCTGCCTCAT							
*TUBα*	Alpha-tubulin	CCATCAAGACCAAGCGCACTA	239	56	94.9	0.999	24.19	0.78	3.27
		CATACCCTCACCCACATACCA							
*TUBβ6*	Beta-tubulin 6	ACTGGGCCAAGGGACACTA	217	56	107.9	0.996	27.39	1.07	4
		GATGGGAACACGGAGAAGG							
*GAPDH*	Glyceraldehyde 3-phosphate dehydrogenase	GCAGAAACCCCGAGGAGATT	250	56	91.1	0.997	25.99	1.61	6.17
		TTAGCAAGAGGAGCAAGGCA							
*HSP90*	Heat shock protein 90	AGAGCAAGATGGAGGAGGTCG	265	56	90	0.998	28.39	1.52	5.36
		AGCAGATGAAAGCAATAAGCA							
*ACT*	Actin	GCCGTGCTTTCCCTCTATG	234	56	95.4	0.999	26.17	0.93	3.61
		GCTTCTCCTTGATGTCCCTTA							
*E2*	Ubiquitin conjugating enzyme 2	CCATCCGAACATCAATAGC	112	56	95	0.997	23.52	1.45	6.15
		CGGTAAGCAGCGAGCA							
*SAMDC*	S-adenosylmethionine decarboxylase	GTTGCTGTGACCATCTTCGGC	291	58	102	0.997	27.32	2.17	7.96
		CTTCTTACCATCCCCCCTCCT							
*PGK*	Phosphoglycerate kinase	GGAAGAGAAGAACGAACC	218	60	107.1	0.996	24.75	2.1	8.47
		CAACAATGGCAGCAAATG							
*PKABA1*	Gene for protein kinase HvPKABA1	AACCATCGGTCACTCAAGC	263	60	101.3	0.998	29.65	2.55	8.59
		CGAGGAGCGACACTACCA							

## Materials and methods

### Plant materials and treatments

The seeds of hulless barley used for this study were purchased from the seed company of Xining City, Qinghai Province in China. After soaking in sterilized water for 16 h, the seeds were first treated with 70% ethanol for 3 min, and surface sterilized with 0.1% HgCl_2_ for 10 min. After fine washing with sterile water five times, the pretreated seeds were placed onto plastic dishes, containing filter paper soaked with sterile water for germination, in an incubator under 100 μE•M^−2^•s^−1^ constant illumination provided via cool white fluorescents at 25°C for 10 days. When the height of seedlings reached 10 cm, the illumination condition was adjusted from constant to a 12/12 h light/dark cycle for 2 days before simulating drought, cold, and salt stress treatments, as an entrainment of the transcriptome-wide gene expression.

For the cold treatment, seedlings were treated for 1 day in the 12/12 h light/dark cycle at 5°C, and then 0.1 g leaf tissues were then harvested every 4 h under constant light. For the drought and salt treatments, seedlings were first cultured in 15% (w/v) PEG6000 (Sangon, China) or 300 mM NaCl (Sangon, China) for 24 h under the 12/12 h light/dark cycle, and then 0.1 g leaf tissues were harvested every 4 h under constant light. All plant samples were immediately frozen in liquid nitrogen and stored at -80°C until RNA extraction.

### Total RNA extraction and cDNA synthesis

Total RNA was extracted from 0.1 g frozen leaf samples via modified Trizol method (TaKaRa, Dalian, China) [49]. Both the quality and purity of these RNAs were assessed via electrophoresis on a 1.0% agarose gel, and quantified via Biospec-nano (SHIMADZU, Japan). All qualified RNA samples with an A_260_/A_230_ ratio of approximately 2.0 and A_260_/A_280_ ratio ranging from 1.9 to 2.1, were first diluted to 1.0 μg/μL and then used as templates for the following reverse transcription experiments. Subsequently, a primeScript^™^ RT reagent Kit with genome DNA Eraser (TaKaRa, Dalian, China) was applied to conduct cDNA synthesizing processes by a recommended procedure, following the instructions of the manufacturer. Following the standard protocol of the manufacturer, cDNAs were purified with a Universal DNA Purification Kit (TIANGEN, China) after which cDNAs were used as templates for following qRT-PCR amplifications.

### Selection and primer design of reference genes

Ten known housekeeping genes from normal barley (*Hordeum vulgare*), a relative species of hulless barley, were selected as candidate reference genes in this study. These genes were *ACT* (GenBank: AY145451.1), *E2* (AY220735), *TUBα* (U40042.1), *TUBβ6* (AM502854.1), *GAPDH* (AK359500.1), *EF-1α* (JN107538.1), *SAMDC* (AK368996.1), *PKABA1* (AB058924.1), *PGK* (AK251528.1), and *HSP90* (AY325266.1). The homologous mRNA sequences of the chosen candidate reference genes were downloaded from the NCBI of the National Institutes of Health via the online Blast tool. According to these mRNA sequences, the primers for qRT-PCR amplification were then designed via software Primer Premier (version 5.0) with the following criteria: the primers should be with limited lengths of 18–25 bp, melting temperatures (Tm) in the range of 50–60°C, GC contents varying from 45 to 55%, and product lengths of 100–300 bp. All primers that have been used in this study were synthesized by Sangon Co. Ltd., in Shanghai, China.

### Semiquantitative RT-PCR and qRT-PCR analysis

The specification and validity of all primers were firstly tested via semiquantitative RT-PCR and the amplifications were triggered by Premix Taq^™^ kits (TaKaRa, Dalian, China). The amplified products of each candidate gene were identified via electrophoresis on a 1.5% agarose gel. The validated PCR primers should account for a single specific amplified product with the correct size.

The quantitative real-time PCR reactions were performed with the Bio-Rad CFX96 Real-Time PCR system (Bio-Rad, USA) using a SYBR^®^ Premix Ex Taq^™^II qPCR kit (TaKaRa, Dalian, China). The qRT-PCR reactions were conducted in mixtures constituted with 12.5 μl 2×SYBR Premix Ex TaqII (TaKaRa, Dalian, China), 0.5 μl of each amplification primer (20 μM), 10.5 μl ddH_2_O, and 1 μl cDNA templates. Each group of reactions was accomplished with three repetitions and one negative control, in which 1 μl ddH_2_O was used instead of cDNA as a template for PCR. After pre-denaturation at 94°C for 30 s, a 2 step-program of qRT-PCR was set as follows: denaturing at 94°C for 5 s, subsequently annealing at 56°C for 30 s, and repeated for 40 cycles. Finally, melting curves of qRT-PCR amplifications were performed to confirm the specificity of the primers again by heating up the products from 56°C to 95°C.

### Analysis of reference gene expression stability

Three public software packages—geNorm, NormFinder, and BestKeeper—were introduced to perform the expression stability analysis of candidate reference genes under various abiotic stresses. When geNorm was used in this analysis, a relative quantity (2^-ΔCt^) of each gene was firstly obtained by calibrating their raw Ct data to the one with the highest Ct value. The normalized Ct values from all tested candidates were then employed to calculate the average M value of test genes [23, 50]. A significant negative correlation was detected between the precise M value of a target gene and its expression stability when assuming 1.5 as critical value for the M value. An M value below 1.5 indicates a confidential candidate for the reference gene; in contrast, an M value above 1.5 has often been used as a rejection criterion for a candidate gene [23]. The smallest M value from geNorm analysis in all examined reference genes always presented the most stably expressed candidate gene. In addition, the pairwise variation (Vn/Vn+1) between two sequential normalization factors can determine the optimal number of reference genes, which needed to be normalized. The recommended cut-off threshold was 0.15, when the pairwise variation was lower than this value, an additional reference gene was not required for the following normalization process. In terms of the software NormFinder, the 2^-ΔCt^ value of each individual gene also served as input data for analyzing gene expression stability. For BestKeeper, the raw data of the Ct value from qRT-PCR was used to calculate the coefficient of variation (CV) and the standard deviation (SD), while the best reference gene was chosen via the lowest CV±SD value. Finally, the appropriate reference genes for qRT-PCR were obtained via combinatory analysis of the results from all three abovementioned algorithms. To verify the reliability of all selected proper reference genes, the relative expression of a known circadian gene, *CIRCADIAN CLOCK ASSOCIATED 1* (*CCA1*), was analyzed in a set of time course samples from various stressed hulless barley leaf tissues. Additionally, a standard curve was first generated from a tenfold gradient dilution of cDNA in a qRT-PCR assay by using the software Microsoft Excel 2003. The efficiency (E) and the regression coefficient (*R*^2^) of the qRT-PCR reactions were then calculated using the slope of the standard curve according to the following equation: E = [10^-(1/slope)^-1]*100%. The average cycle threshold (Ct) values from three independent biological repetitions were used to carry out the statistical analysis of the target genes' relative expression, while data from each of the biological repetitions was presented by three technical repeats.

## Results

### Genetic information of candidate reference genes for qRT-PCR

A total of the ten most commonly used reference genes in the reports of qRT-PCR in graminaceous crops, including *ACT*, *E2*, *TUBα*, *TUBβ6*, *GAPDH*, *EF-1α*, *SAMDC*, *PKABA1*, *PGK*, and *HSP90*, were chosen as candidates for gene expression stability assessment under various abiotic stresses (cold stress, salt stress, and drought stress) The specification of primers for all candidate genes was verified via PCR amplification before performing the qRT-PCR reactions. Only those ones that could trigger an amplification of identical products with predicted size were identified as qualified primers (S1 Fig). Subsequently, the performances of these chosen primers were further verified by observing their melting curves during qRT-PCR, and a single sharp peak in the melting curves always presented high quality primers (S2 Fig). The qRT-PCR products of all tested genes in this study ranged from 112 bp to 293 bp. The efficiencies of each qRT-PCR reaction of all candidate reference genes were above 90% and varied from 90% to 107.9% with egression coefficients, denoted as R^2^, distributed from 0.996 to 0.999.

**Fig 1:**
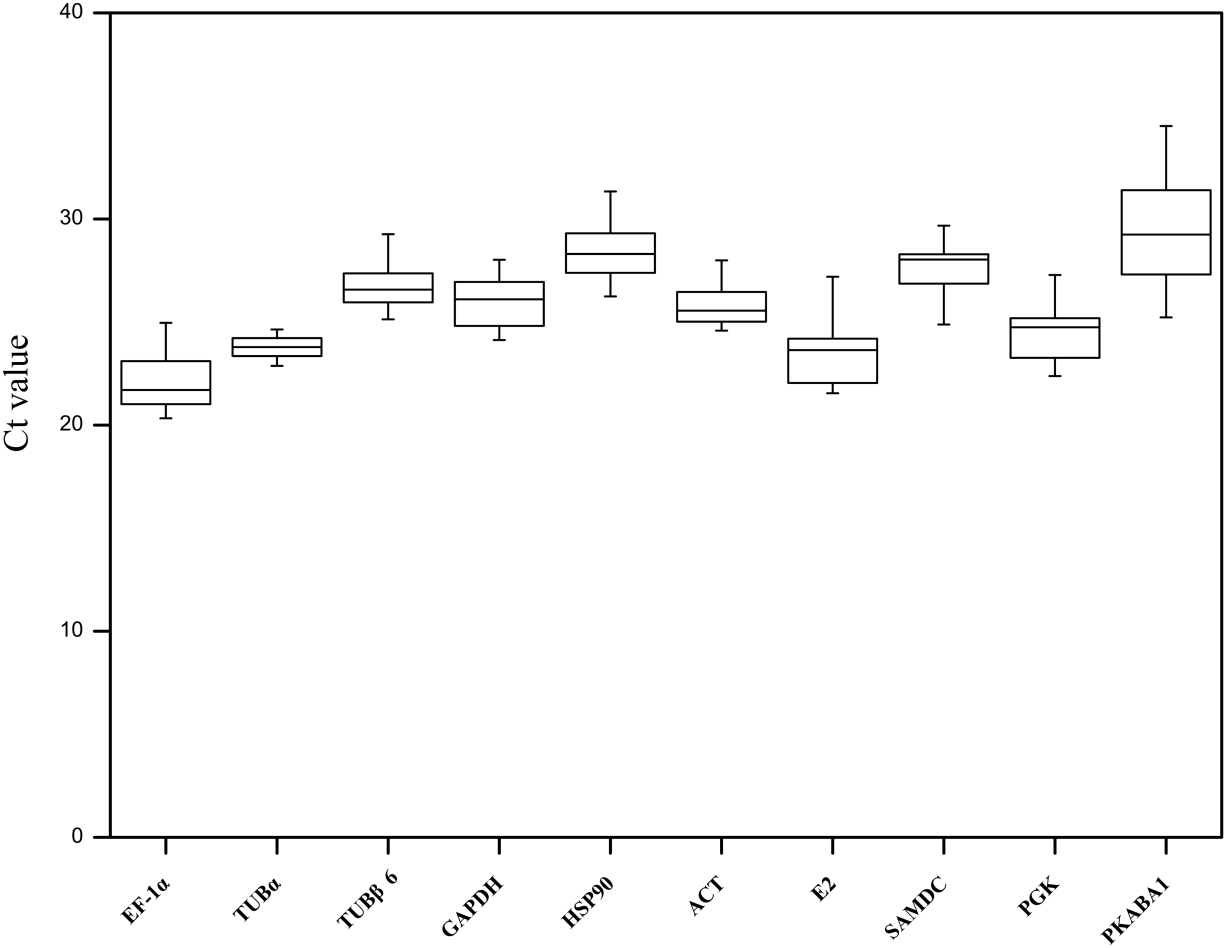
Cycle threshold (Ct) values of ten candidate reference genes across all experimental samples. The Ct value of each gene shows the mean of three biological replicates. Box graph indicates the interquartile range. The line across the box depicts the median. The lower and upper dashes are determined by the 25^th^ and the 75^th^ percentiles.

To analyze the expression level of these 10 candidate reference genes under three different experimental conditions, the Ct values of all analyzed samples were obtained via qRT-PCR, and the average Ct values of each gene under all experimental groups were calculated. The results revealed that the Ct value of 10 candidate reference genes varied from 22.30 to 29.63 (Fig 1), and the most abundant expression gene was *EF-1α* with the lowest average Ct ± SD (22.34±1.78), followed by *E2*, *TUBα*, *PGK*, *ACT*, *GAPDH*, *TUBβ6*, *SAMDC*, *HSP90*, and *PKABA1* (Table 1). *PKABA1* was the gene with the lowest expression level, but the highest average Ct ± SD (29.65±2.55). The SD values of *TUBα* and *ACT* were minimal (24.19±0.78 and 26.17±0.93), indicating that they shared the smallest variation of the ten candidate genes. The coefficient of variation (CV) of the Ct values also represents the stability of gene expression. In other words, a smaller CV value of the reference gene indicated a more stable expression. Among the ten candidate reference genes, the CV values of *TUBα* (3.27%) and *ACT* (3.61%) had the lowest variation, while the *PKABA1* (8.59%) had the highest across all tested samples (Table 1). In summary, these results show that the expression abundance of candidate reference genes under different experimental conditions has played a pivotal role in the screening of appropriate reference genes in Tibetan hulless barley.

### geNorm analysis

**Fig 2:**
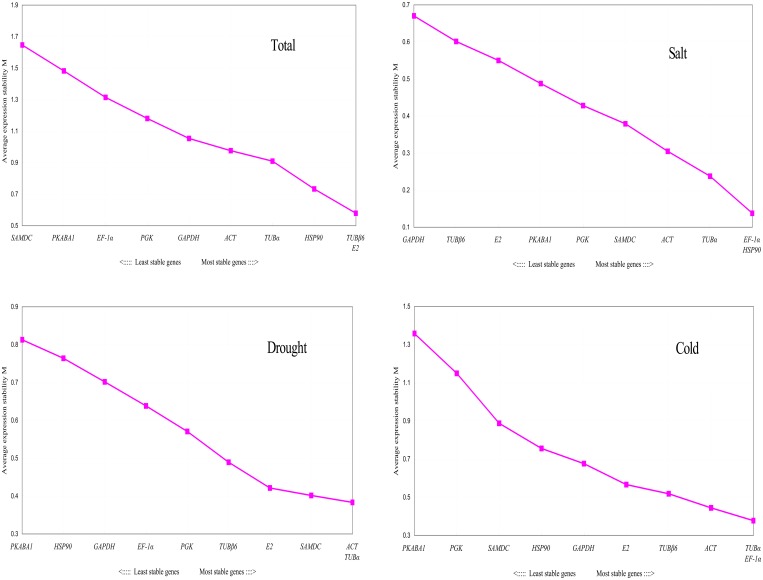
Expression stability values of 10 candidate reference genes evaluated via geNorm. The most stable reference genes are arranged on the right lines in these graphs, while the least stable genes are placed on the left, following their ranks of the expression stability among all tested reference genes under various stresses.

The software geNorm determines the ranking of the ten candidate reference genes by calculating the average pairwise expression ratios (presented as M) of each candidate reference gene. The M value of the gene is negatively related to its stability, while 1.5 is definitively configured as the threshold of the M value. If the M value of a candidate gene is below 1.5, this gene can be used as a confident reference gene, otherwise it should be rejected for use as a reference gene. The results of this present study reveal that the M values of all tested candidate reference genes were below 1.5 either under drought, cold, or salt stressed conditions. However, when combining the data for all the samples from three stressed conditions, the M value of *SAMDC* and *PKABA1* were larger than 1.5. In summary, it could be confirmed that the M value of the selected candidates in this study were relatively smaller, demonstrating that these reference genes are relatively stable under individual stress environments. Although the different performances of individual genes have been detected under various stressed conditions in general, the *TUBβ6* and *E2* with the lowest M value were found to be the most stable reference genes under all tested situations, while the genes of *SAMDC*, *PKABA1*, and *PGK* were the least stable. Concerning of the situation of various stress treatments, the two best reference genes for samples from the salt stress condition were *EF-1α* and *HSP90*. For the drought stress conditions, *ACT* and *TUBα* were the most stable reference genes. The most suitable reference genes under cold stress were *TUBα* and *EF-1α* (Fig 2).

**Fig 3:**
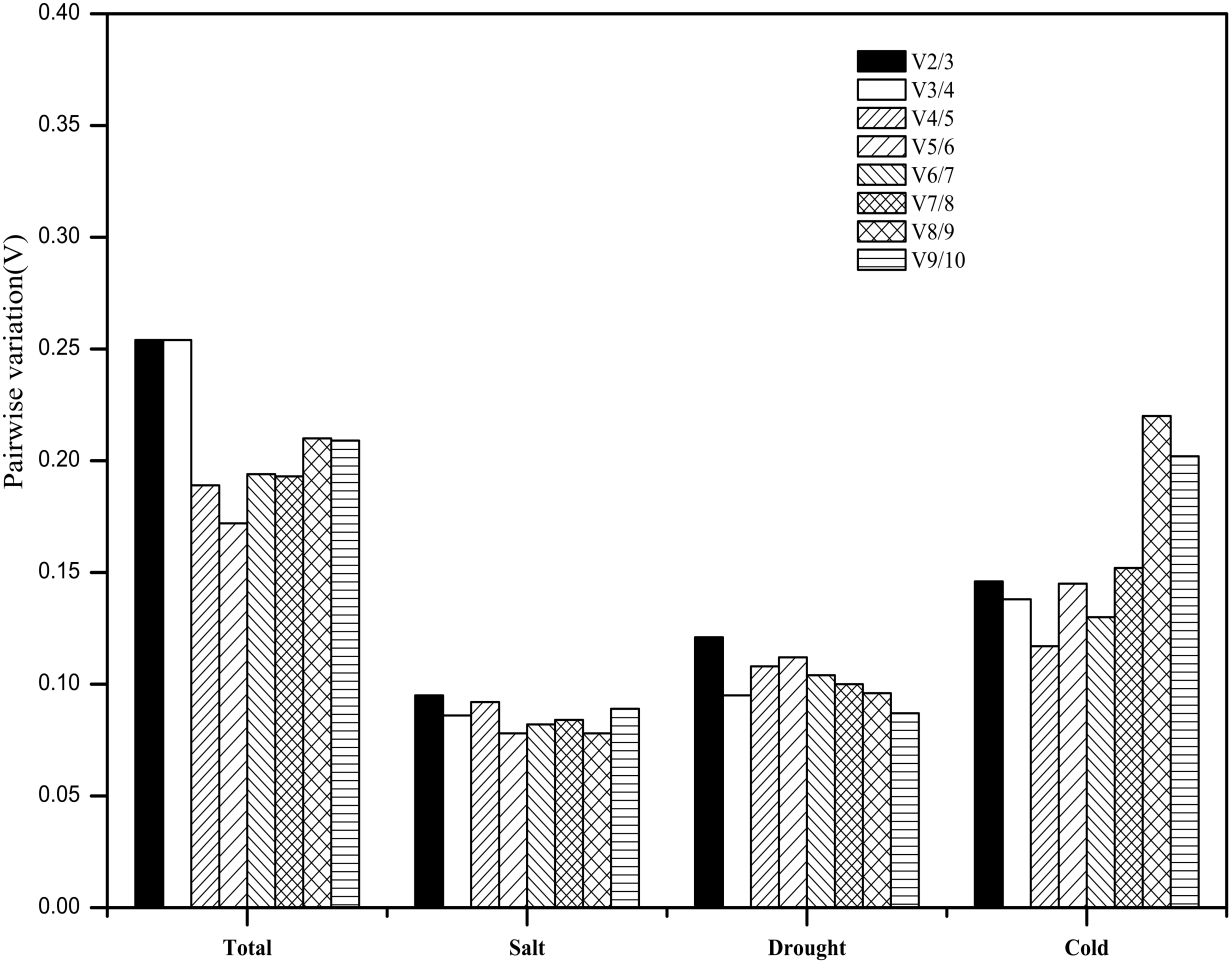
Pairwise variation (V) of 10 candidate reference genes under various stresses calculated via geNorm to determine the optimal number of reference genes for normalization. Pairwise variation (Vn/n+1) was calculated between the normalization factors (NFn and NFn+1) with the geNorm program to determine the optimal number of reference genes for qRT-PCR data normalization of various samples.

Subsequently, by analyzing the pairwise variation (Vn/Vn+1) of 10 candidate reference genes, the optimal number of reference genes was determined under different stress treatments. As described in Fig 3, the V2/3 values from the salt stress, drought stress, and cold stress groups were below 0.15 (0.095, 0.121, and 0.146, respectively), signifying that only two reference genes—*EF-1α* and *HSP90* for salt stress, *ACT* and *TUBα* for drought stress, and *TUBα* and *EF-1α* for cold stress—were required to calibrate the expression of target genes under each stress condition. When all stresses were considered, the V5/6 value (0.172) was the lowest; however, it was still above the threshold of 0.15. This result indicates that no tested reference genes could be generally used to normalize the expression of target genes under the three tested stress conditions.

### NormFinder analysis

The rank of expression stability of the candidate reference genes via NormFinder was determined according to the stability values of each reference gene. Lower stability data collected by this software represented higher expression stability of the candidates. Under the salt stress conditions, *HSP90* and *TUBα* displayed most stable genes expression, while *GAPDH* exhibited the least stability. For cold stress, the most stably expressed reference genes were *E2* and *TUBβ6*. In contrast, the *PKABA1* gene, which had been newly used as a reference gene in some reports for barley proved to be the least stable under cold stress. When the general performance of the candidates under all tested stresses was considered, *GAPDH* and *TUBβ6* were the most suitable reference genes for qRT-PCR via NormFinder, although *TUBβ6* and *GAPDH* were ranked ninth and tenth, respectively for the salt stress condition. Under drought stress subset, *TUBβ6* and *GAPDH* were ranked seventh and eighth, respectively. Simultaneously, the data of Table 2 also shows that *TUBβ6* and *GAPDH* are ranked second and fourth, respectively, under cold the stress condition. These results indicate that the stability of individual reference genes exhibits enormous variations under different stresses. As shown in Fig 2 and Table 2, for salt stress, drought stress, and the combined results from three tested stresses, no significant differences were identified between the results from the evaluation of expression stabilities of these candidate reference genes, which were conducted either via the software NormFinder or via geNorm methods. However, under cold stress, the hierarchy of gene expression stability analysis performed by NormFinder was distinctly different from the results produced by geNorm. The best reference genes, *TUBα* and *EF-1α*, that were recommended by geNorm were located as the sixth and seventh in all 10 tested candidates sorted by NormFinder.

**Table 2:** Expression stability of candidate reference genes calculated by NormFinder.

Rank	Total		Salt		Drought		Cold	
	Gene	Stability	Gene	Stability	Gene	Stability	Gene	Stablity
1	*GAPDH*	0.423	*HSP90*	0.087	*E2*	0.053	*E2*	0.112
2	*TUBβ6*	0.470	*TUBα*	0.123	*ACT*	0.272	*TUBβ6*	0.186
3	*E2*	0.490	*ACT*	0.164	*TUBα*	0.332	*HSP90*	0.311
4	*HSP90*	0.552	*EF-1α*	0.171	*PGK*	0.338	*GAPDH*	0.347
5	*TUBα*	0.568	*PGK*	0.234	*SAMDC*	0.381	*ACT*	0.517
6	*EF-1α*	0.823	*SAMDC*	0.325	*EF-1α*	0.416	*EF-1α*	0.618
7	*ACT*	0.851	*PKABA1*	0.378	*TUBβ6*	0.450	*TUBα*	0.710
8	*PGK*	1.012	*E2*	0.455	*GAPDH*	0.510	*SAMDC*	0.792
9	*PKABA1*	1.260	*TUBβ6*	0.533	*HSP90*	0.542	*PGK*	1.289
10	*SAMDC*	1.427	*GAPDH*	0.607	*PKABA1*	0.588	*PKABA1*	1.386

### BestKeeper analysis

The rank of the expression stability of reference genes via BestKeeper was determined according to the CV±SD of their average Ct values (Table 3). Synthesizing all the data from various sample subsets, two genes with the lowest CV±SD values, 2.28±0.54 for *TUBα* and 2.89±0.74 for *ACT*, were confirmed to be the most stable reference genes. Individually, under the salt stress condition, the *TUBβ6* and *E2* genes showed lowest CV±SD values with 1.17±0.3 and 1.38±0.3, and were ranked as the top two stable reference genes. Similar to this, the results from the samples of drought stressed subset indicated that the *TUBβ6* (with a CV±SD value of 1.91±0.53) and *E2* (with a value of 3.15±0.77) genes were the most stable genes. Furthermore, in the cold stress sample subsets, *TUBα* (with a CV±SD value of 1.6±0.38) and *EF-1α* (with a CV±SD value of 2±0.42) were chosen to be the most stable reference genes by the software BestKeeper.

**Table 3:** Expression stability of candidate reference genes calculated via BestKeeper.

Rank	Total			Salt			Drought			Cold		
	Gene	SD	CV	Gene	SD	CV	Gene	SD	CV	Gene	SD	CV
1	*TUBα*	0.54	2.28	*TUBβ6*	0.30	1.17	*TUBβ6*	0.53	1.91	*TUBα*	0.38	1.60
2	*ACT*	0.74	2.89	*E2*	0.30	1.38	*E2*	0.77	3.15	*EF-1α*	0.42	2.00
3	*TUBβ6*	0.89	3.31	*PGK*	0.39	1.69	*TUBα*	0.77	3.19	*ACT*	0.47	1.80
4	*E2*	1.10	4.66	*TUBα*	0.41	1.75	*ACT*	0.79	3.13	*TUBβ6*	0.63	2.31
5	*HSP90*	1.20	4.20	*HSP90*	0.41	1.53	*HSP90*	0.88	3.00	*E2*	0.75	3.09
6	*GAPDH*	1.23	4.70	*EF-1α*	0.41	1.90	*PKABA1*	0.89	2.74	*GAPDH*	0.93	3.59
7	*PGK*	1.27	5.15	*ACT*	0.45	1.79	*EF-1α*	0.98	4.01	*PGK*	1.10	4.32
8	*EF-1α*	1.41	6.33	*SAMDC*	0.46	1.65	*PGK*	0.98	3.82	*HSP90*	1.10	3.77
9	*SAMDC*	1.61	5.90	*PKABA1*	0.76	2.74	*SAMDC*	0.98	3.40	*SAMDC*	1.54	6.19
10	*PKABA1*	2.20	7.43	*GAPDH*	0.96	3.83	*GAPDH*	1.12	4.09	*PKABA1*	1.84	6.41

### Comprehensive stability analysis of reference genes

To get more reliable stably expressed reference genes, comprehensive ranking (Table 4) of stability for reference genes was often integrated by the results from three open access software packages [51]. This strategy has also been used in the present study. As shown in Table 5, the most and least stable combinations of reference genes were based on geNorm, NormFinder, and BestKeeper. In terms of total sample subsets, *TUBβ6*, *E2*, *HSP90*, and *TUBα* were ranked as the most stable reference genes, while *HSP90* and *TUBα* were confirmed to be the optimal stable reference genes in the salt stress subset. Furthermore, *TUBα* and *EF-1α* were the most stable reference genes in the cold stress subset. Moreover, the data of the drought stress subset indicated that the *ACT* and *E2* genes were the most stable genes. Interestingly, although *PKABA1* has been used as a novel reference gene by some studies on the detection and quantifying of nucleic acid molecules in wheat and barley [39], the results of this study demonstrated that the Ct values of *PKABA1* under various stress conditions were too large to be acceptable. This reflects that the expression of *PKABA1* has been down-regulated by environmental stress conditions. Accounting for the lower abundance of its mRNA in cells of Tibetan hulless barley, the ranks of *PKABA1* are on the rear part of the list for the expression stability of candidate reference genes under various stresses from all three software packages.

**Table 4:** Expression stability ranking of the 10 candidate reference genes.

Method	1	2	3	4	5	6	7	8	9	10
**(A) RANKING ORDER UNDER ALL SAMPLES (BETTER-GOOD-AVERAGE)**										
geNorm	*TUBβ6*	*E2*	*HSP90*	*TUBα*	*ACT*	*GAPDH*	*PGK*	*EF-1α*	*PKABA1*	*SAMDC*
NormFinder	*GAPDH*	*TUBβ6*	*E2*	*HSP90*	*TUBα*	*EF-1α*	*ACT*	*PGK*	*PKABA1*	*SAMDC*
BestKeeper	*TUBα*	*ACT*	*TUBβ6*	*E2*	*HSP90*	*GAPDH*	*PGK*	*EF-1α*	*SAMDC*	*PKABA1*
Comprehensive ranking	*TUBβ6*	*E2*	*TUBα*	*HSP90*	*GAPDH*	*ACT*	*PGK*	*EF-1α*	*PKABA1*	*SAMDC*
**(B) RANKING ORDER UNDER SALT STRESS (BETTER-GOOD-AVERAGE)**										
geNorm	*EF-1α*	*HSP90*	*TUBα*	*ACT*	*SAMDC*	*PGK*	*PKABA1*	*E2*	*TUBβ6*	*GAPDH*
NormFinder	*HSP90*	*TUBα*	*ACT*	*EF-1α*	*PGK*	*SAMDC*	*PKABA1*	*E2*	*TUBβ6*	*GAPDH*
BestKeeper	*TUBβ6*	*E2*	*PGK*	*TUBα*	*HSP90*	*EF-1α*	*ACT*	*SAMDC*	*PKABA1*	*GAPDH*
Comprehensive ranking	*HSP90*	*TUBα*	*EF-1α*	*ACT*	*PGK*	*E2*	*TUBβ6*	*SAMDC*	*PKABA1*	*GAPDH*
**(C) RANKING ORDER UNDER DROUGHT STRESS (BETTER-GOOD-AVERAGE)**										
geNorm	*ACT*	*TUBα*	*SAMDC*	*E2*	*TUBβ6*	*PGK*	*EF-1α*	*GAPDH*	*HSP90*	*PKABA1*
NormFinder	*E2*	*ACT*	*TUBα*	*PGK*	*SAMDC*	*EF-1α*	*TUBβ6*	*GAPDH*	*HSP90*	*PKABA1*
BestKeeper	*TUBβ6*	*E2*	*TUBα*	*ACT*	*HSP90*	*PKABA1*	*EF-1α*	*PGK*	*SAMDC*	*GAPDH*
Comprehensive ranking	*ACT*	*E2*	*TUBα*	*TUBβ6*	*SAMDC*	*PGK*	*EF-1α*	*HSP90*	*GAPDH*	*PKABA1*
**(D) RANKING ORDER UNDER COLD STRESS (BETTER-GOOD-AVERAGE)**										
geNorm	*TUBα*	*EF-1α*	*ACT*	*TUBβ6*	*E2*	*GAPDH*	*HSP90*	*SAMDC*	*PGK*	*PKABA1*
NormFinder	*E2*	*TUBβ6*	*HSP90*	*GAPDH*	*ACT*	*EF-1α*	*TUBα*	*SAMDC*	*PGK*	*PKABA1*
BestKeeper	*TUBα*	*EF-1α*	*ACT*	*TUBβ6*	*E2*	*GAPDH*	*PGK*	*HSP90*	*SAMDC*	*PKABA1*
Comprehensive ranking	*TUBα*	*EF-1α*	*TUBβ6*	*ACT*	*E2*	*GAPDH*	*HSP90*	*PGK*	*SAMDC*	*PKABA1*

**Table 5:** Comprehensive results of selected suitable reference genes based on geNorm, NormFinder, and BestKeeper.

Total		Salt		Drought		Cold	
Most	Least	Most	Least	Most	Least	Most	Least
*TUBβ6*	*SAMDC*	*HSP90*	*GAPDH*	*ACT*	*PKABA1*	*TUBα*	*PKABA1*
*E2*		*TUBα*		*E2*		*EF-1α*	
*TUBα*							
*HSP90*							

### Validation test of the chosen reference genes

**Fig 4:**
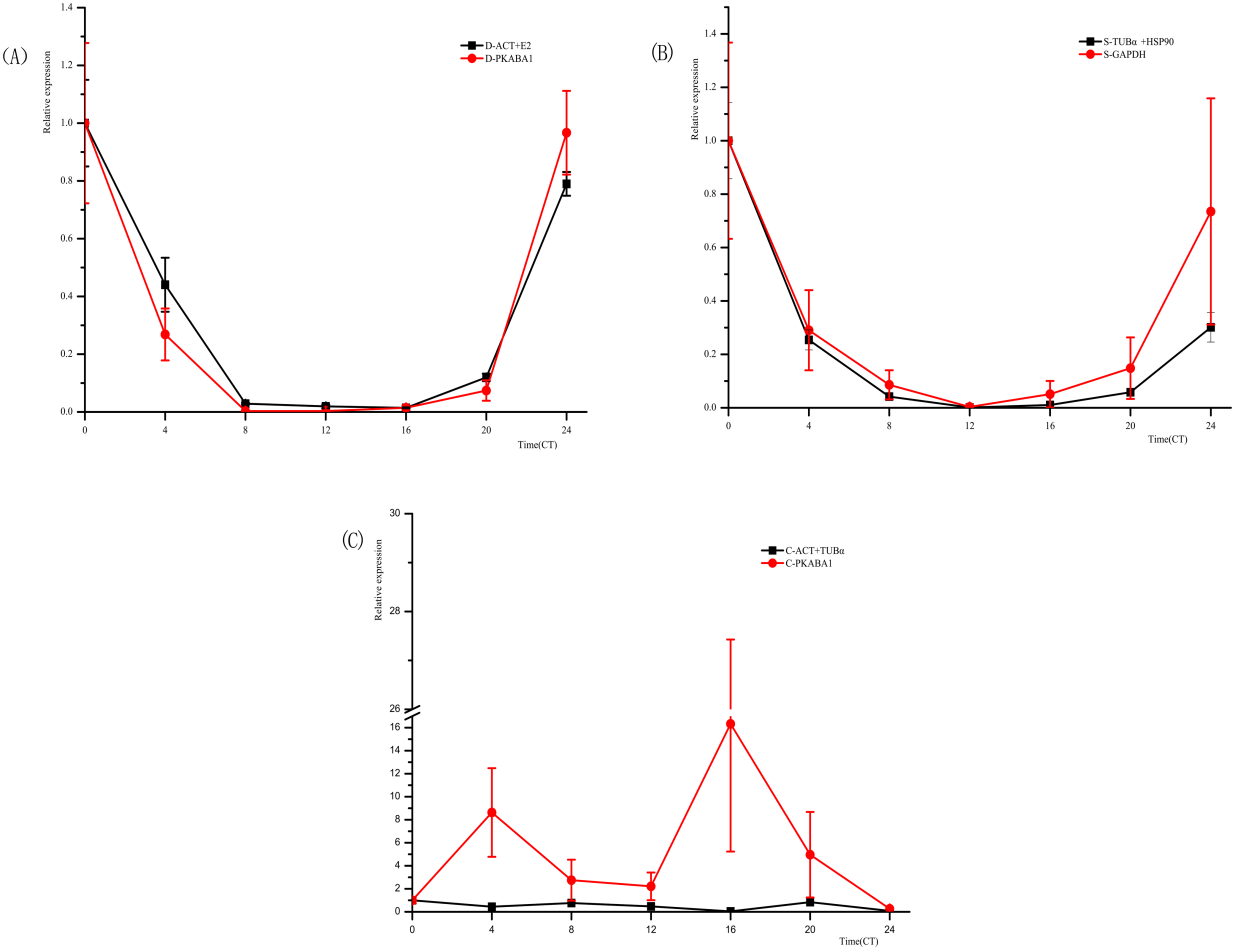
Normalized expression level of *CCA1* using validated reference genes. Samples were collected from10-day-old seedlings treated by drought, salt, and cold stress for 0, 4, 8, 12, 16, 20, and 24 h under LL conditions. (A), (B), and (C) represent the relative expression of *CCA1* under drought, salt, and cold stress conditions, respectively. Error bars indicate standard errors.

To validate the suitability of candidates, the reference genes that have been ranked as the best or the worst were used to calibrate the expression of the target gene *CIRCADIAN CLOCK ASSOCIATED 1*, under the stress conditions of drought, salt, and cold. Serving as one of the most important morning-phased components in the circadian rhythmic controlling system of higher plants, CCA1 is a MYB-like transcription factor with enormous abundance in the morning phase [52, 53]. As shown in Fig 4, the expression profile of *CCA1* was analyzed with the qRT-PCR method under drought stress condition, while the optimized two reference genes *ACT* and *E2* were used as references. The results are consistent with the Alabadı’s report in Arabidopsis, where the expression peak of *CCA1* was observed at time point CT_0_, and the trough was located at CT_12_ [45]. Although similar results also can be achieved with the *PKABA1* gene (the least suitable candidate), which was used as a reference gene, the SD value of *CCA1* expression data normalized via *PKABA1* was too large to be accepted. This result showed that the data calibrated by *PKABA1* was not as stable as the data calibrated by *ACT* and *E2*. Furthermore, the average cycle threshold (Ct) values of the *PKABA1* gene in certain stress samples varied around 30, which was much higher than that of other candidate reference genes. This phenomenon indicated that the abundance of *PKABA1* mRNA in the transcriptome of drought stressed hulless barley was too low to meet the primary requirement of a qualified candidate gene. In the salt stress subset, positive results were obtained via software, recommending the optimal genes *TUBa* and *HSP90* as references to analyze the relative expression of *CCA1*. While negative results were detected for *GAPDH* (a rejected reference gene judged by software), this was used to calibrate the expression of *CCA1* with unacceptable SD values and disturbed qRT-PCR results. The risk of generating unrealistic results during data normalization processes has rendered the *GAPDH* gene unsuitable as a reference for analyzing the expression of target genes under salt stress condition. Similarly, when *PKABA1* was used to calibrate the expression of *CCA1* under cold stress, large SD values and two unexpected distinct peaks located at false time points in the relative expression profiles of *CCA1* were found in 24 h time-course samples stressed by low temperature. This result is inconsistent with a report by Seo et al. for Arabidopsis [54]. In contrast, when the two optimal reference genes *TUBα* and *EF-1α* were used as references under the same cold stressed condition, results consistent with a previous report were obtained [54]. In summary, *PKABA1* is not a suitable reference gene to normalize the expression of the target gene under cold and drought stress conditions.

### Discussion

The expression pattern of luxury genes under a particular environment is one of the most important features of the target genes during the identification of their functions. Northern blotting, competitive RT-PCR, microarray, and qRT-PCR are the methods generally used for gene expression pattern detection. However, when the accuracy, sensitivity, specificity, and efficiency of these methods are concerned, qRT-PCR was the optimal choice for a small amount of genes in large sample pools. The selection of appropriate reference genes is essential for the performance of qRT-PCR reactions. Ideally, the reference genes should express stably under various environmental conditions, across different developmental stages, or in individual tissues. Before the large-scale sequencing methods were widely applied in the RNA studies, some so-called housekeeping genes were used by researchers as reference genes, such as *EF-1α*, *UBQ*, *CYP*, *ACT*, and *TUB*. The translated products of most of these genes were involved in controlling and carrying out some basic metabolic activities in the cell, or maintaining the primary structures of the cell skeleton system. However, an increasing number of studies have shown that the expression of these abovementioned housekeeping genes are not as constant as they were once assumed to be, especially under various environmental stress conditions [55]. These revelations have negatively affected the reliability of the results of many qRT-PCR studies in which housekeeping genes were used as references without optimization. Reliable reference genes have played a pivotal role during the process of gene expression evaluation via qRT-PCR. In recent years, Carvalho *et al*. have reported that the two optimized stable expressed housekeeping genes *EF-1α* and *ADP* and a commonly used reference gene *TUB* were used to calibrate the expression of a same target luxury gene, *P5CS*, in a comparative study. The *EF-1α* and *ADP* genes were selected and evaluated with the software geNorm, where the *TUB* gene was not obtained from the software analysis, but from previous reports where it was most commonly used as a reference for qRT-PCR in the studies from *Citrus* species. The results of Carvalho’s study demonstrated that the expression profiles of the target gene, were rather similar when the raw expression data from *P5CS* had been normalized by the *EF-1α* and *ADP* as references; however, significant differences were detected between this and the expression data of the target gene from the same set of RNA samples only when *TUB* was introduced as the reference gene [56]. This indicates that it is necessary to assess and analyze the stability of candidate reference genes before the implementation of qRT-PCR reactions. For this purpose, various software packages have been developed to conduct the tasks of evaluating the stability of reference genes. GeNorm, NormFinder, and BestKeeper were the most widely used programs [42–44, 57, 58].

Abiotic stresses, such as drought stress, salt stress, or extreme temperatures, always remarkably limited the growth and yield of plants. Studies concerning how those abiotic stresses influenced the growth of plants have been one of the predominant issues for plant sciences research. In particular, many researchers have focused on the identification and characteristics of plant resistance genes to abiotic stresses or the breeding of new plant germplasm resources with significantly promoted abiotic stress resistance, either via genetic engineering or cellular engineering methods. The selection of the most stable reference genes under different stressed conditions would provide a reliable basis for the accurate analysis of resistance gene expression.

In terms of the expression stability of reference genes under low temperature stress, diverse results have been obtained in different plant species. Studies from *cucumber* have shown that the expression of both *CYP* and *ACT2* were the most stable reference genes under cold stress [59]. However, some other reports from *S*. *sibiricum*, tomato, and rice indicated that the *EF-1α* gene was the most stable reference gene under cold stress condition [38, 40, 60]. In the present study, the results showed that *TUBα* and *EF-1α* were the most suitable reference genes for hulless barley under low temperature stress when both stability and repeatability were concerned. Generally consistent with the abovementioned reports by Hong et al., Løvdal and Jain, found that the comprehensive rank of *EF-1α* is located at the second place in the list of the expression stability of all 10 candidate reference genes tested under cold treatment [38, 40, 60]. Therefore, *EF-lα* can be used as a reference gene for qRT-PCR in hulless barley under low temperature stress. In addition, we also found that *TUBα* is a more stable reference gene than *EF-lα*, which was reported as the most unstable reference gene in *Lycoris aurea* under cold stress subset [61]. In contrast, the results from our present work showed that the *TUBα* was ranked first place in the list of the expression stability of all 10 candidate reference genes tested under cold treatment. As far as drought stress condition was concerned, the results from our study revealed that *ACT* and *E2* were also the two candidate reference genes with top expression stability in hulless barley. In contrast to studies in maize under drought stress, *TUBβ* and *EF-1α* were confirmed as the best reference genes, while *GAPDH* and *ACT2* ranked third and fifth, respectively [62]. Our results from hulless barley have demonstrated that *ACT* and *E2* were the most stable reference genes, while *TUBβ6*, *EF-lα*, and *GAPDH* ranked fourth, seventh, and ninth place on the list, respectively. In summary, it can be concluded that *ACT* and *E2* are competent reference genes for qRT-PCR tests under drought stress.

Under the salt stress condition, distinct qualified reference genes have been used to analyze gene expression in different plant species. The studies in *Oxytropis ochrocephala Bunge* grown under salt stress indicated that *GAPDH2*, *HIS*, and *ACT101* were the top three reference genes with highest stability, while *GAPDH1* and *18S* were the least stable reference genes [63]. A report in Jute (*Corchoruscapsularis*) revealed that *RAN*, *ACT7*, and *EF-lα* were the top three stable reference genes, while *18S rRNA* and *ACT* were the least stable reference genes under salt stress [51]. This is contrary to results by Hua et al. [14], which have reported that *ACT2*, *UPL*, and *TIP41* were the most stable reference genes in barley under abiotic stress [14]. Results of the present reference gene screening work in hulless barley convinced that *HSP90* and *TUBα* were the two most competent candidates with top stability, followed by *EF-lα*, *ACT*, *E2*, *PGK*, *TUBβ6*, *SAMDC*, *PKABA1*, and *GAPDH* with gradient dropped stability.

It is quite difficult to find an identically optimal reference gene that is universally suited to evaluate gene expressions in plants in different environments or diverse growth stages that is verified by a large body of evidence from either different plant species under various abiotic stress, or the same species of plant but in different developmental stages [50, 64, 65]. This might be partially attributable to differences among the categories of reference genes that have been selected for stability detections in separated research reports [66, 67]. However, by employing different types of software (using various weighted stability parameters from qRT-PCR data via different algorithms), the stability of certain groups of candidate reference genes could be evaluated in a combinative manner. The optimized reference genes selected with this method can be used to calibrate the expression of individual target genes under various conditions with significantly promoted accuracy and efficiency [68, 69].

## Conclusion

Three different statistical algorithms were used to study the stability of 10 candidate reference genes in hulless barley under abiotic stress conditions. The results showed that under the salt stress condition, the most stable expression of the reference genes were *HSP90* and *TUBα*, while *GAPDH* had the worst stability. Meanwhile, the best stable reference genes were *ACT*, *E2* and *TUBα*, *EF-1α*, respectively, at the drought and cold stress conditions. The most stable reference genes screened in this work can efficiently improve the accuracy and standardization of the expression of target genes under abiotic stress conditions by qRT-PCR analysis in Tibetan hulless barley.

## Supporting information

S1 Fig Agarose gel (1.5%) electrophoresis shown the amplification of a single PCR product with expected size. M represents 2000 bp DNA marker.
(TIF)

S2 Fig Melting curves of 10 candidate reference genes shown single peaks in qRT-PCR. (TIF)
