# Supplementary figures and images for: Selection of appropriate reference genes for the detection of rhythmic gene expression via quantitative real-time PCR in Tibetan hulless barley
Source: PLoS One. 2018 Jan 8;13(1):e0190559. doi: 10.1371/journal.pone.0190559 (PMC5757941; doi:10.1371/journal.pone.0190559)

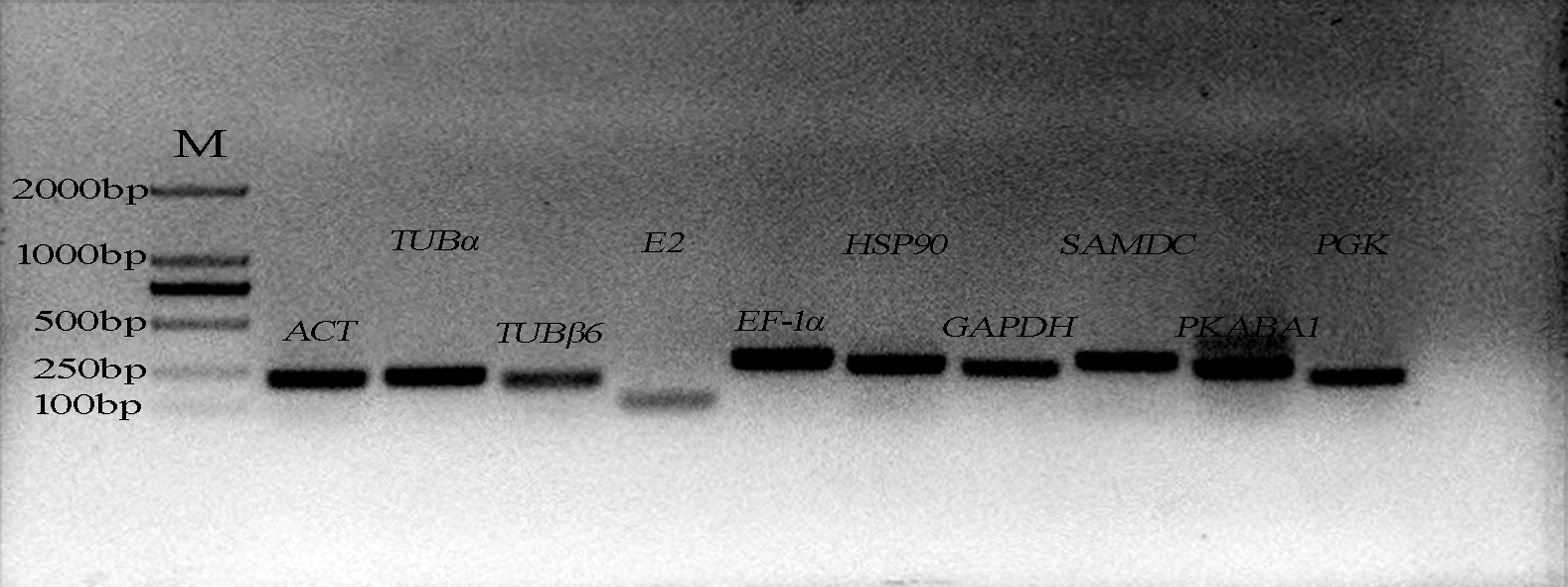

Supplement: S1 Fig — M represents 2000 bp DNA marker. (TIF) [file pone.0190559.s001.tif]

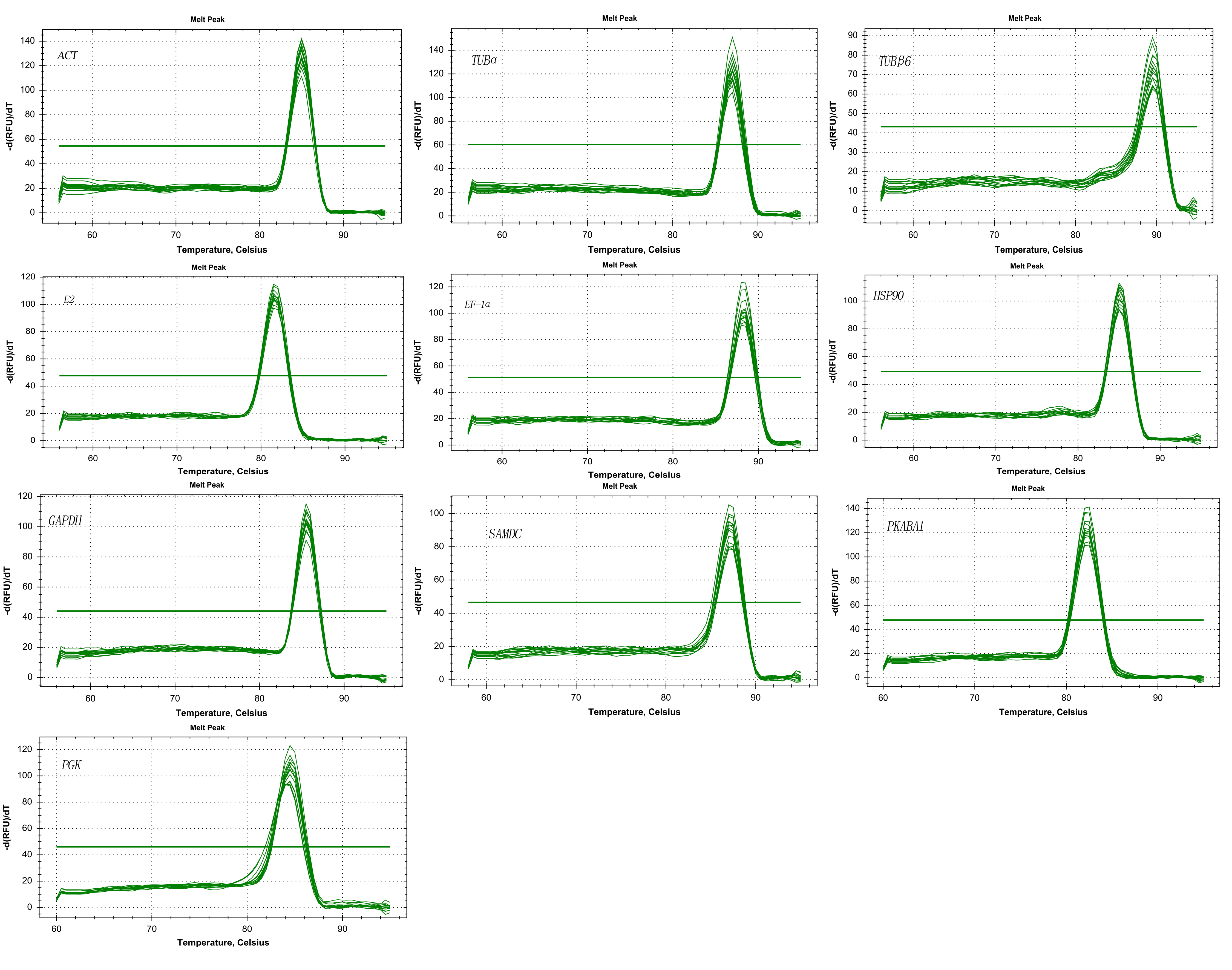

Supplement: S2 Fig — (TIF) [file pone.0190559.s002.tif]
